# Selection of right medical students to combat rural shortage of doctors: could it be a solution? In perspective of Bangladesh

**DOI:** 10.15694/mep.2021.000161.1

**Published:** 2021-06-07

**Authors:** Laxmi Saha, Arjun Chandra Dey, Humayun Kabir Talukder

**Affiliations:** 1 King Hamad University Hospital; 2 Centre for Medical Education

**Keywords:** Rural retention, physicians shortage, selection process, physicians background

## Abstract

This article was migrated. The article was marked as recommended.

This comparative cross-sectional study was conducted to find the association between duration of service in rural health facilities and physicians’ background factors to redress geographic imbalances in physician distribution. Among 6898 participants, information of 989 were retrieved from Directorate General of Health Services (DGHS), Minsitry of Health and Family Welfare (MOHFW), Bangladesh, through systematic sampling. Physicians who worked in rural health facilities for less than 3 years were labelled as group A, and those worked 3 years or more in rural places were put in group B. Background factors of two groups were compared and proportion of doctors living and working in rural areas was sorted. Among the participants, eighty percent were working in urban facilities and 50% worked in rural areas for less than three years. Proportion of females was about 30% and there was no significant differences between male and female in terms of duration of stay in rural areas in both groups (p=0.07). The association between place of completion of secondary school certificate (SSC) examination and duration of services in the rural areas were found statistically significant (p=0.003). Apart from this, no other background factors were found to be significantly associated.

## Introduction

Geographically skewed distribution of health personnel, with a higher proportion of physicians in urban and wealthier areas ( Rabinowitz, 2001; Dussault and Franceschini, 2006) is a worldwide, longstanding, and serious problem ( Wilkinson *et al.,* 2009) in health sectors. Fewer doctors work in areas with a large population, compared to a large number of doctors clustered in areas of less population, as described by Hart’s ‘Inverse Care Law’ ( Wright and Jablonowski, 1987). This inequity is particularly severe in developing countries such as Bangladesh, where about 70% of the population live in rural areas ( Darkwa *et al.,* 2015), in contrast, less than 20% of the doctors practice in those places ( Rawal *et al.,* 2015). Likewise, it was reported that only 11 doctors per 100,000 population in rural areas, compared to 182 per 100,000 in urban areas in the country ( Ahmed *et al.,* 2013). This is one of the greatest challenges in achieving ‘access to quality essential health care services for all’, a key component of Sustainable Development Goals (SDGs). Therefore, it is crucial to ensure equal distribution of health work forces including graduate doctors in rural areas ( WHO, 2018).

Researchers found rural exposure to education, recreation, and upbringing facilitates rural practice of doctors in the future ( Dixit, 2002; Lindelow and Serneels, 2005; Besley and Ghatak, 2005). However other authors commented this association as inconclusive ( Hancock *et al.*, 2009). To overcome the problem of rural shortage of health work forces, World Health Organization issued evidence-based guidelines on rural retention in 4 major domains: education, regulatory, financial incentives, and professional-personal support ( WHO, 2010). Many more economically developed countries (MEDC) reviewed different factors and programs to improve this imbalance and found rural background of medical students as a positive factor to choose rural areas as future working places ( Brooks *et al.,* 2002; Ross, MacGregor and Campbell, 2015). However, rural areas of MEDC are different from that of less economically developed countries (LEDC). Hence, to solve this long-standing problem in Bangladesh, it is important to find out whether there is any association of doctors’ background factors and preference of working in rural areas. This comparative cross-sectional study was conducted with the aim to find the association between government doctors’ background and duration of work in rural facilities. Findings would explore information regarding doctors who are more likely to work in rural areas which may guide the selection process of medical students in the future.

## Methods

The study was carried out from July 2018 to June 2019 by retrieving information of doctors from Management Information Systems (MIS) of Directorate General Health Service (DGHS) (See Supplementary File 1), Bangladesh. Information of 6898 doctors who had been working in the government sector up to 2015 were entered in the sampling frame. Among them 1300 subjects were selected randomly by systematic sampling with an interval of 6 from the sampling frame. After exclusion of missing data, finally 989 doctors records met the inclusion criteria. Missing data of the study was 23.9% of the sample.

In terms of location, primary health care centers named Union Sub-Centre and Upazila Health Complex (UHC) were taken as rural areas, whereas secondary and tertiary health facilities including district and divisional hospitals were considered as urban facilities. In this study, doctors who worked less than 3 years in rural health facilities were taken as group A, and those who worked in rural areas 3 years or more were included in group B for comparison. The information of doctors included age, gender, places of grade 10 school board examination (Secondary School Certificate, SSC), grade 12 higher secondary school certificate (HSC) examination (See Supplementary File 1), duration of service in rural and urban facilities, current working place and living area. The required data were retrieved from the doctors’ record of MIS of DGHS and plotted in a spreadsheet. In case of any missing data, the respective doctors were contacted over telephone, and disagreement to provide information resulted in exclusion from the study.

Quantitative data were analyzed by SPSS 20 using p < 0.05 as level of significance differences between variables. Chi-square analysis was done for categorical variables. The odds ratio (OR) was determined to find the association between background factors of the doctors and duration of stay in rural health facilities.

## Results/Analysis

Among 989 doctors, 71% were male; 72% had their Secondary School Certificate (SSC) in urban areas, and 97% completed Higher Secondary School Certificate (HSC) in urban areas. Sixty two percent of the participants completed their post graduation. In the study, only 8% of the doctors lived in rural areas and about one fifth (19.7%) were working in rural facilities of the country Table 1.

**Table 1:** Distribution of the characteristics of government doctors included in the study

Characteristics	Frequency	Percent
**Gender**		
Male	702	70.97
Female	287	29.3
**Current Address**		
Rural	79	8
Urban	910	92
**Post-graduation**		
Yes	615	62.4
No	370	37.6
**Place of SSC**		
Rural	229	23
Urban	760	77
**Place of HSC**		
Rural	32	3.2
Urban	957	96.8
**Current work place**		
Rural	183	19.7
Urban	732	78.7
Other	15	1.6

**Figure 1.**
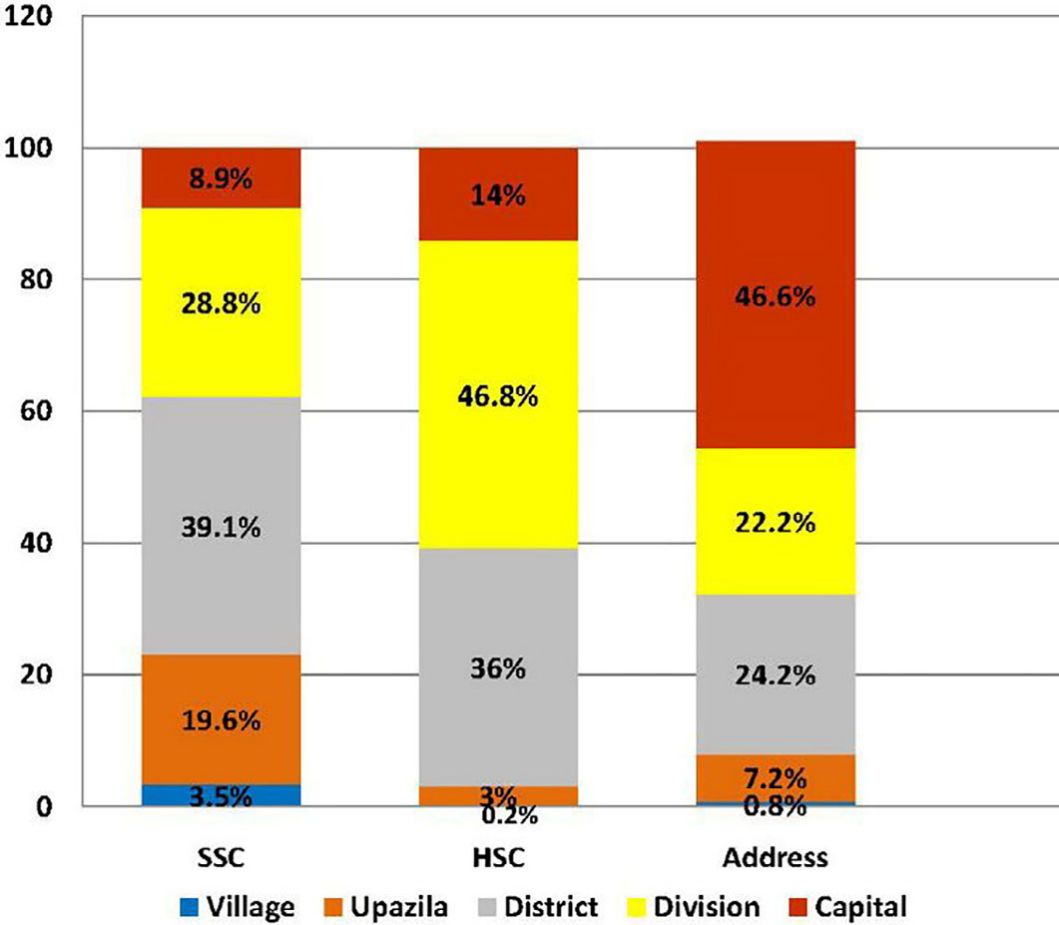
Place of education and living areas according to the administrative units of Bangladesh

Figure 1 illustrates the place of education and living areas according to the administrative units of Bangladesh (See Supplementary File 1). Two hundred and twenty-nine (23%) doctors completed their SSC from rural areas, whereas 77% did it from urban areas with major portion in district school (39%). In regard to HSC, only 32 (3%) doctors completed from rural schools with great majority (97%) from the urban areas, divisional schools being the largest contribution (46.8%). Regarding living areas, only 79 (8%) doctors were in rural areas whereas about 451 (46%) stayed in the capital city of the country.

**Figure 2.**
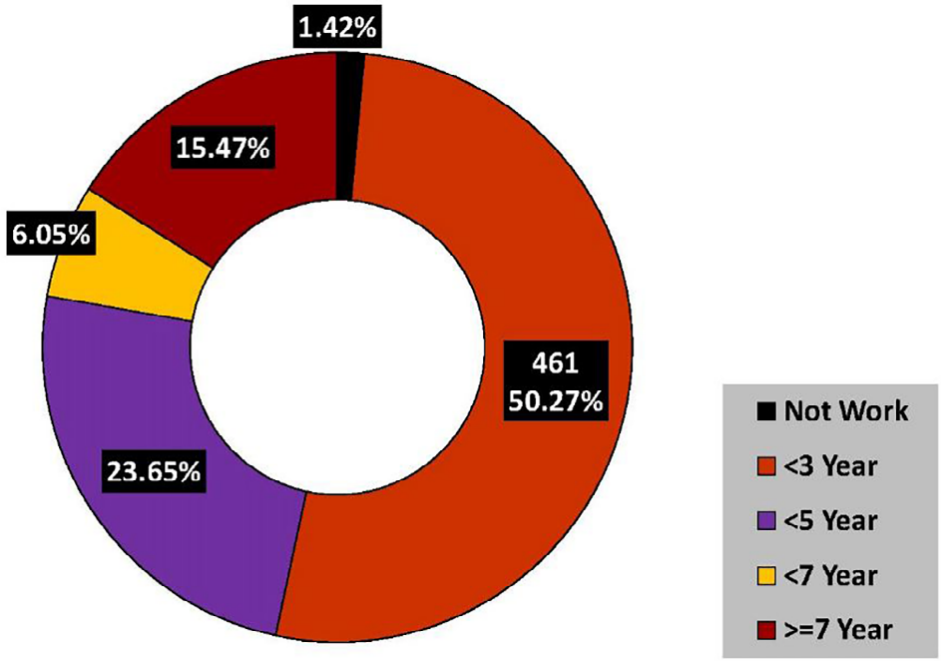
Duration of work in the rural facilities

Among participants, 892 (90%) completed their 5 years in government service, 461 (50.27%) of them worked in the rural areas for less than 3 year. Thirteen (1.42%) doctors never served in rural areas. One hundred fifty-three (15.47%) of them were posted in rural areas for 7 years or more ( Figure 2).

The association of duration of service in rural areas to gender and place of education were compared ( Table 2). Group A consisted approximately 47% of the study participants and group B contained 44%. In group A, about 69% were male, while in group B, 73% were male. No significant association was found between gender and duration of work in rural set up (p=0.07). In group A, 79.82% had SSC exam from urban institutes, compared to 70% in group B. The association between place of SSC and work duration in rural areas were statistically significant (p=0.003) in two groups. In both the groups, very high proportion of doctors (98.26% and 94.43%) had their HSC done from urban schools. No significant association was found between the place of HSC and duration of work in rural areas (p=0.08).

**Table 2:** Association of gender, educational place with working place

Characteristics of the participants	Group A n (%)	Group B n (%)	P value	OR	CI
**Gender**					
Male	318 (68.98)	315 (73.08)	0.07	0.79	0.59-1.05
Female	143 (31.01)	114 (26.45)			
**Total N (%)**	461 (46.61)	431(43.58)			
**Place of SSC examination**					
Rural	92 (19.96)	126 (29.23)	0.003	0.64	0.47-0.89
urban	368 (79.82)	304 (70.53)			
**Place of HSC examination**					
rural	07 (1.5)	23 (5.33)	0.08	0.54	0.25-1.15
urban	453 (98.26)	407 (94.43)			

## Discussion

This study clearly indicates that most doctors in the study population grew up (approx. 80%), living (90%) and working (80%) in urban areas. With these urban dominant health care providers, it is questionable how they can be retained in rural areas.

The government of Bangladesh has taken initiatives to address the issues of rural shortage of doctors and to improve the health care facilities to the rural communities. Among them, few steps are as follows:

Primary Health Care (PHC) infrastructure: In Bangladesh, Upazila [sub-district] Health Complex (UHCs) and Union Subcenter are considered as Primary Health Care (PHC) facilities and were designed to provide a wide range of healthcare functions. Despite well designed PHC infrastructure in the public sector, physician shortage in underserved areas is a decade long problem in Bangladesh and only 25% of government doctors are working in rural facilities ( Ahmed *et al.,* 2011). In this study, the findings are similar, about 20% of the study population had worked in PHC.

Compulsory rural service of medical doctors: Since the 1980s, the compulsory service of doctors in rural health care facilities has been implemented by the Government. According to the revised gazette of Transfer and Posting Policy for Officers in Health Service ( Joarder *et al.*, 2018), the newly appointed doctors must serve at least two years in rural areas. However, despite having this rule in place, half of the study participants (50%) served in rural health facilities less than 3 years. It means presumably the existing policy is not helping to improve the situation.

Encouraging rural doctors to post-graduation: Government doctors who worked in remote areas for at least one year, get a chance to enroll in a post-graduation course, whereas without working in a remote area the time limit is at least 2 years ( BSMMU, 2021).

Reserved district quota for medical admission: For the country’s medical admission, 20% quota are reserved for district students which are selected by parents’ district of origin, rather than where they were raised. This policy was adopted long ago when communication to and living standards in district levels were less developed than present time. Therefore, this policy may not be conducive to the students who live in rural areas in the present context.

Many countries, including developed and developing, tried different programs to encourage students with rural background for medical education to solve the problem and found positive association with rural retention of physicians in the future ( Budhathoki, Zwanikken and Pokharel, 2017; Verma *et al.,* 2016.). This study results also found that doctors who spent their school life in rural areas had worked longer in primary health facilities. This association was statistically significant (p = 0.03). Considering this study findings, authorities of medical education could think about changing the entry criteria of medical students to encourage more rural students in the selection process. However, questions may come about how students with a rural background will be selected. Bangladesh is a plain land with some hilly areas where all upazila (sub-district) and villages are not similar in terms of infrastructure and facilities. For example, understandably suburban places adjacent to metropolitan cities are more developed than similar suburbs of smaller towns. Therefore, it is important to find a way to select the genuine students who were raised in the districts to avail the facility of reserve seats for those areas. In these circumstances, the place of SSC can be considered as the place of raising.

Another important finding of the study was fewer number of female doctors working in government sector (30%) but nosignificant association between duration of work in rural health facilities and gender (p=0.07). This indicates femalegender is probably not a negative factor for rural staying. Lack of or less interest to work by female doctors in the government sector is not a new problem; in 2007, there was a gross imbalance in gender ratio, favoring males in the service, four males to one female ( Ahmed *et al.,* 2011). Therefore, the policymakers need to consider the female students with rural backgrounds who, in the future, are more likely to stay in rural places.

This study revealed that more than half of the study participants (62.4%) did their post graduation, hence intended specialist career rather than general practitioner. In Bangladesh, 2-3 years post graduate training is a mandatory requirement, before taking the exit post graduate examination. Therefore, medical education can implement mandatory rural training, at least for some duration, as a requirement for postgraduate training. This might rectify to some extent in alleviating the problem. At the same time, this will help them to be familiar with the community and its related health problems. However, study with underserved areas postgraduate training found mixed outcomes. Comparative study in USA observed rural training reduced rural shortage whereas an Australian study without having comparison, found a small percentage (14%) of individuals reported that they were influenced against rural practice after their placements ( Verma *et al.,* 2016). Therefore, the effect of rural post-graduate training needs further evaluation.

Overall, the study revealed that despite several steps taken by the Government of Bangladesh, there is ongoing severe shortage of physicians in rural and remote health facilities. The problem will not resolve unless evidence-based steps are taken for the change of attitude and motivation of doctors. In fact, success or failure in attracting physicians to rural facilities or retaining them in rural posts vastly depends on their choice of working places, not only on the policy.

In Bangladesh, most of the previous studies regarding this subject were conducted by taking interviews of medical students to find their future preferences of workplaces and analysis of ongoing government policies to improve the condition. As future workplace choices are ever evolving and complex, those studies could assume rather than find the association of rural staying of doctors and related factors. This study was conducted by taking information from doctors on the job. It reflected the realistic scenario of physicians’ choice and the situation of the health system in Bangladesh. Therefore, the findings of the study might be helpful in assessing the association between rural stay and background factors of physicians.

This study had limitations as missing data was nearly 24% due to retrospective collection of information which might have affected some of the findings. The study worked on only two background factors of participants. As many other complex matters are involved in rural retention of doctors, prospective studies with large samples should be conducted to explore the impact of other factors like, curriculum, spouse motivation, facilities, monitoring, logistic and infrastructural support etc. Moreover, the study included only government doctors which may not be reflective of the total scenario of the country though the private health sector of Bangladesh too is predominantly urban based ( Dussault and Franceschini, 2006).

## Conclusion

In summary, the study revealed that the health care system of Bangladesh is dominantly urban based whereas most of its population live in rural areas. The proportion of doctors working in rural facilities were substantially less. Number of female doctors in the government sector is low as well. However, there was no statistically significant association in gender and duration of stay in rural facilities. In contrast, upbringing in rural places was significantly related to duration of service in rural health facilities. Most of the doctors preferred specialization rather than working as general practitioners. It can be concluded that, Bangladesh should come out from the urban based health care system to ensure health services to its majority of population. Encouraging more students from rural areas, implementing post-graduate training facility in underserved areas, ensuring suitable environment and policy for retaining more female doctors in rural places, are the possible additions to be considered in the health policy. Addressing the rural shortage followed by monitoring of sustainability to achieve the desired outcome in this regard would be the key in overall improvement of the condition.

## Take Home Messages

• Medical course selection criteria need to be modified to encourage more rural students.
• Place of secondary school should be considered as place of origin to meet district quota.
• Female gender is not a negative factor for rural retention.
• Training in rural facilities should be included in post graduate training.
• More prospective studies are required to solve this complex problem.

## Notes On Contributors

**Laxmi Saha**, a specialist Obstetricians and Gynaecologist is also a Master in Medical Education. Among her publications the most remarkable one titled, ‘Use of in vitro Fertilisation Prediction Model in an Asian Population-Experience in Singapore’ which was awarded as the best merit award of Annals of Medicine, Singapore 2015.

**Arjun Chandra Dey** is a Consultant Neonatologist at King Hamad University Hospital with a teaching experience of 18 years. He has 15 publications so far to his credit. In addition he is working as Senior Clinical Lecturer at Royal College of Surgeons Ireland (RCSI), Bahrain Campus.

**Md. Humayun Kabir Talukder** is working as Professor of Curriculum Development & Evaluation at Centre for Medical Education, Dhaka, Bangladesh. He is one of the pioneer medical educationists in Bangladesh. He has a number of publications related to medical education in various international journals.
