# Supplementary material for: Selection of right medical students to combat rural shortage of doctors: could it be a solution? In perspective of Bangladesh
Source: MedEdPublish (2016). 2021 Jun 7;10:161. [Version 1] doi: 10.15694/mep.2021.000161.1 (PMC10939572; doi:10.15694/mep.2021.000161.1)
Supplement: Peer review history

# Reviewer report: Reviewer response for version 1

**DOI:** 10.21956/mep.18901.r27005

**Published:** 2021-07-09

**Authors:** Richard Hays

**Affiliations:** 1 James Cook University

This review has been migrated. The reviewer awarded 4 stars out of 5 I agree that the influence of possible predictors of rural practice location is an important research topic outside of the developed nations, where more is known. My experience has taught me that the meaning of 'rural' differs widely between nations. There are many measures within local definitions, but all include population data and proxies for access to services, particularly in education and health care. In Australia, Canada and the USA there is strong overlap with being 'remote' in a geographic sense, elsewhere large rivers and mountain ranges are more important. What we have in common is that there is a consistent urbanisation with major lifestyle changes that seem to make working in larger centres more attractive. There is also an international trend away from primary care careers and relatively few narrower specialty positions outside of urban centres, making workforce research very complex. In this study, we see that rurality (defined by address at graduation from secondary school) is a predictor of future practice. This supports policies that actively recruit rural background students into health professional courses. It would be worth increasing intake of rural background students and following up to see if this improves the workforce distribution, although the impact is likely to be stronger with additional rural curriculum, assessment, clinical placement experience and role modelling, as found in Australian research. The main weakness of this paper is the low response rate, but it is still worth reading for rural educators both in Bangladesh and elsewhere, as it strengthens the international evidence base.

Reviewer Expertise:

NA

No decision status is available

# Reviewer report: Reviewer response for version 1

**DOI:** 10.21956/mep.18901.r27004

**Published:** 2021-06-08

**Authors:** Jennifer Cleland

**Affiliations:** 1 University of Aberdeen

This review has been migrated. The reviewer awarded 4 stars out of 5 It is really important to have papers reporting on workforce issues from less developed countries and I applaud the authors in their efforts to bring some understanding of the issues in Bangladesh to a wide audience. They are correct in saying that the patterns seen on more developed countries may not be the same as those in less developed ones. However, given the findings, it would have been nice to have have included comparison with previous literature, particularly from Puddey and Playford in Australia, as well as studies from Canada/NOSM. How this issue is addressed does indeed need changes in policy and selection criteria and practices. Remote and rural exposure, role models from remote and rural areas as teachers and other curricular strategies also need to be considered.

Reviewer Expertise:

NA

No decision status is available
